# Supplementary material for: Executive Functions of Adults with Binge-Eating Disorder: The Role of Weight Status and Psychopathology
Source: Brain Sci. 2021 Dec 22;12(1):6. doi: 10.3390/brainsci12010006 (PMC8773845; doi:10.3390/brainsci12010006)
Supplement: Supplementary file 1 [file brainsci-12-00006-s001.zip › brainsci-1464803-supplementary.pdf]

**Supplementary Materials for**

Nele Busch, Ricarda Schmidt, Anja Hilbert

# Executive Functions of Adults with Binge-Eating Disorder: The Role of Weight Status and Psychopathology

**Supplementary Table:** Linear Regression Analyses Using Clinical Parameters as Predictors and General and Food-Specific Executive Function Measures as Criteria.

**Supplementary Figures:** Number of Participants Included in Multivariate Analyses of Variance in Measures of General and Food-Specific Executive Functions.

**Table S1.** Linear Regression Analyses Using Clinical Parameters as Predictors and General and Food-Specific Executive Function Measures as Criteria.

| Measures | BMI | | | | PHQ-9 sum score | | | | EDE-Q global score | | | |
|---|---|---|---|---|---|---|---|---|---|---|---|---|
| | *n* | β | *p* | $R^2$ | *n* | β | *p* | $R^2$ | *n* | β | *p* | $R^2$ |
| Cards and Lottery Task | | | | | | | | | | | | |
| NAD *T* score | 72 | 0.074 | .539 | .005 | 69 | −0.088 | .472 | .008 | 69 | −0.028 | .817 | .001 |
| Stop-Signal Task | | | | | | | | | | | | |
| SSRT *T* score | 70 | 0.062 | .612 | .004 | 67 | −0.274 | .025 | .075 | 67 | −0.190 | .124 | .036 |
| Go/No-Go Task | | | | | | | | | | | | |
| Commission errors *T* score | 76 | −0.039 | .736 | .002 | 73 | −0.138 | .244 | .019 | 73 | 0.028 | .817 | .001 |
| N-Back Verbal | | | | | | | | | | | | |
| Correct responses *T* score | 76 | 0.058 | .621 | .003 | 73 | −0.128 | .279 | .017 | 73 | −0.032 | .790 | .001 |
| Trail Making Test | | | | | | | | | | | | |
| Completion time *T* score | 75 | −0.077 | .511 | .006 | 72 | −0.129 | .281 | .017 | 72 | −0.154 | .198 | .024 |
| Tower of London | | | | | | | | | | | | |
| Planning score *T* score | 70 | −0.127 | .294 | .016 | 67 | −0.155 | .210 | .024 | 67 | −0.066 | .593 | .004 |
| WAFA intrinsic visual | | | | | | | | | | | | |
| Mean RT *T* score | 75 | 0.138 | .239 | .019 | 72 | −0.226 | .056 | .051 | 72 | −0.067 | .575 | .005 |
| Food-specific DPT | | | | | | | | | | | | |
| Food attentional bias *T* score | 73 | 0.078 | .510 | .006 | 70 | −0.057 | .641 | .003 | 70 | −0.071 | .559 | .005 |
| Food-specific AAT | | | | | | | | | | | | |
| Approach bias high-caloric *T* score | 62 | 0.107 | .406 | .012 | 61 | 0.058 | .660 | .003 | 61 | 0.131 | .314 | .017 |

Note. AAT = Approach Avoidance Task; BMI = body mass index; DPT = Dot Probe Task; EDE-Q = Eating Disorder Examination-Questionnaire; NAD = number of advantageous decisions; PHQ-9 = Patient Health Questionnaire depression module; RT = reaction time; SSRT = stop-signal reaction time; WAFA = "Perception and Attention Functions Battery Alertness".
β represents the standardized regression weight.
Due to multiple testing, significance level was adjusted to $\alpha = .0056$ using Bonferroni correction.

**Figure S1.** Number of Participants Included in Multivariate Analyses of Variance in Measures of General Executive Functions.

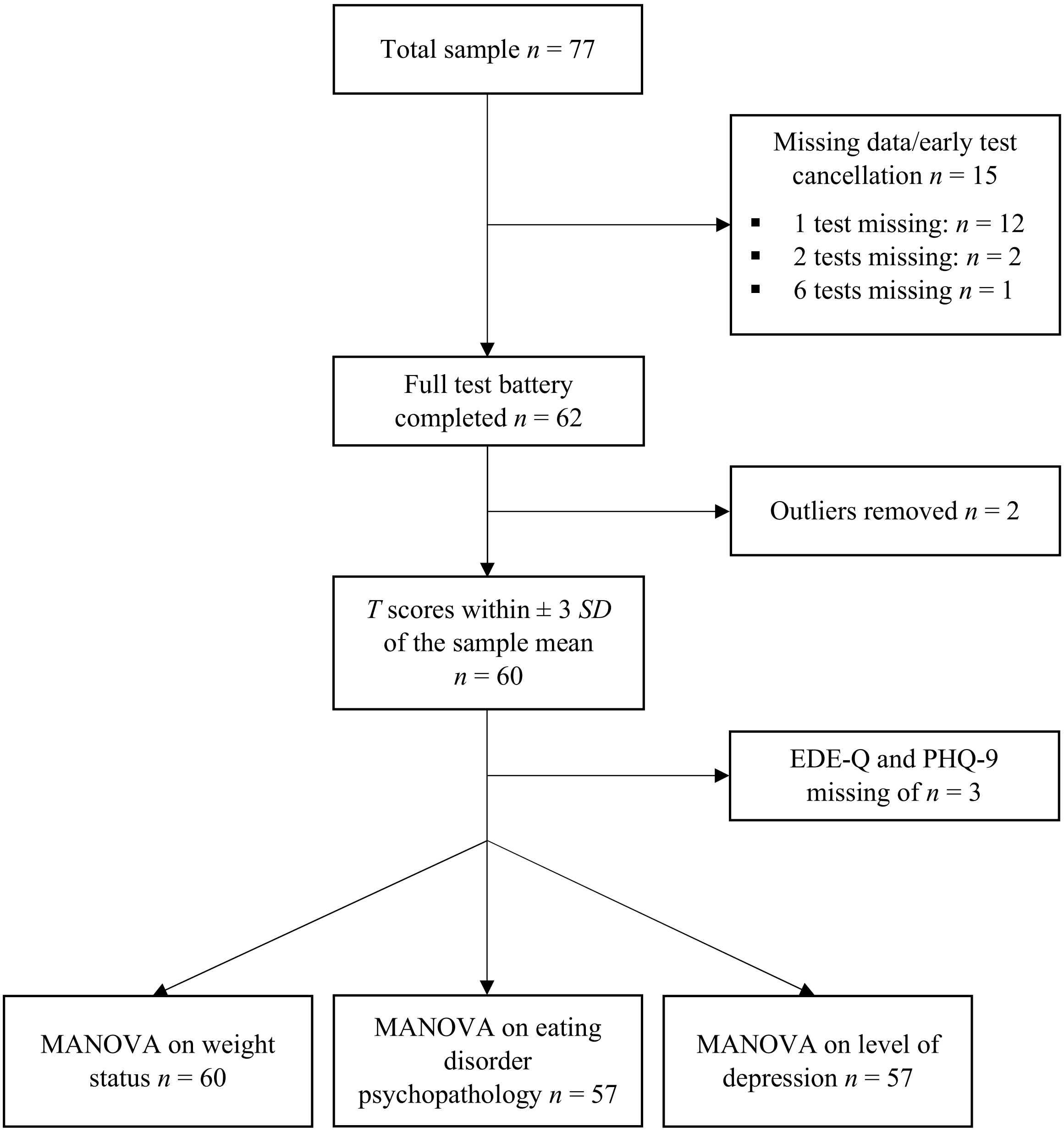


Note. EDE-Q = Eating Disorder Examination-Questionnaire; MANOVA = multivariate analysis of variance; PHQ-9 = Patient Health Questionnaire depression module.

**Figure S2.** Number of Participants Included in Multivariate Analyses of Variance in Measures of Food-Specific Executive Functions.

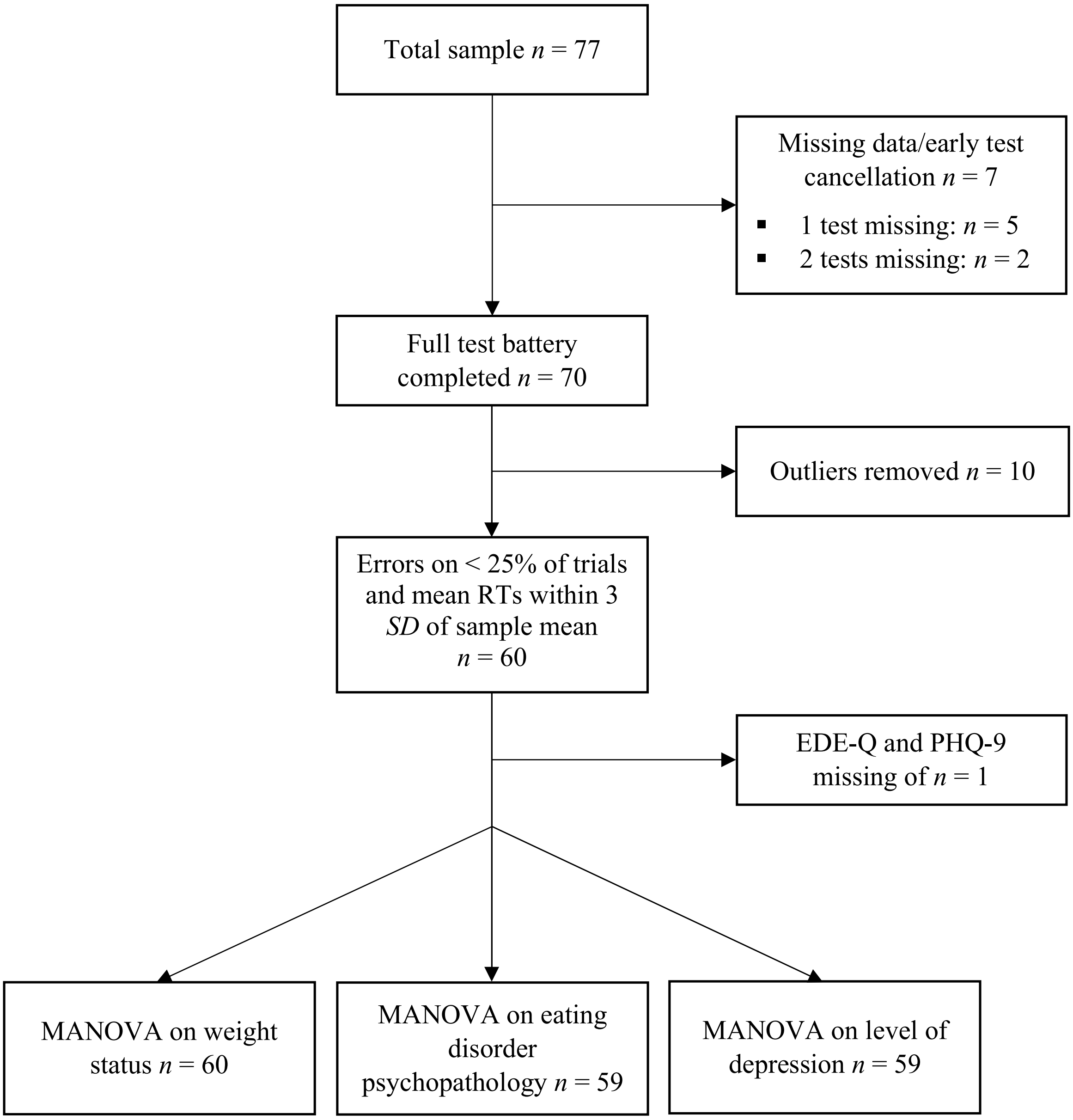


Note. EDE-Q = Eating Disorder Examination-Questionnaire; MANOVA = multivariate analysis of variance; PHQ-9 = Patient Health Questionnaire depression module.
